# Supplementary material for: Healing of Ocular Herpetic Disease Following Treatment With an Engineered FGF-1 Is Associated With Increased Corneal Anti-Inflammatory M2 Macrophages
Source: Front Immunol. 2021 May 13;12:673763. doi: 10.3389/fimmu.2021.673763 (PMC8158292; doi:10.3389/fimmu.2021.673763)
Supplement: Supplementary file 1 [file DataSheet_1.pdf]

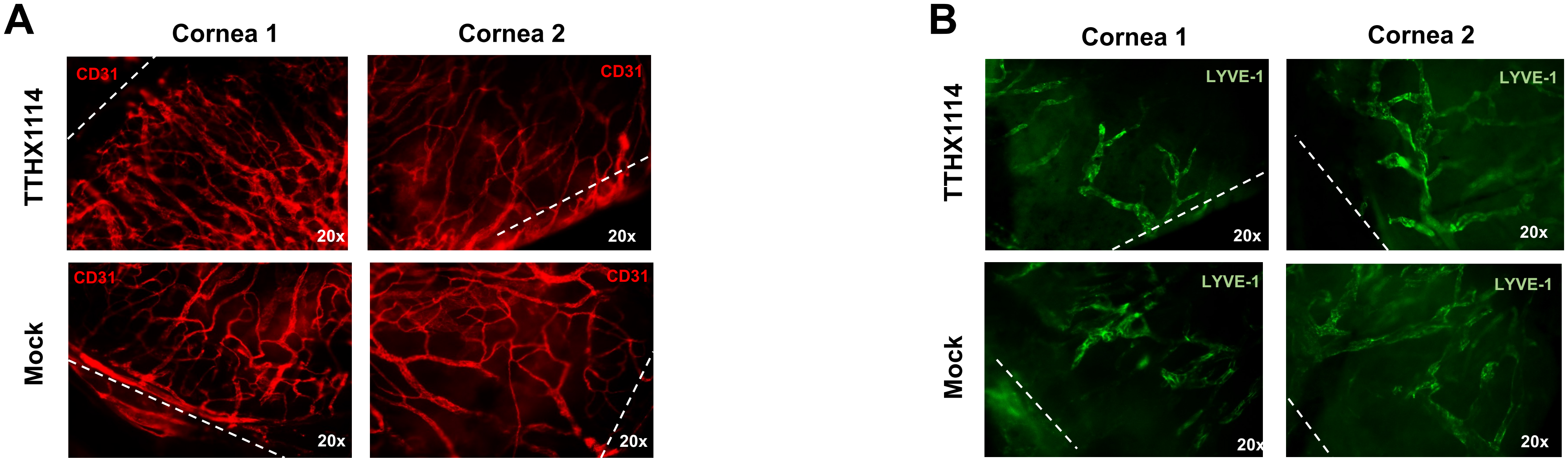

<u>**Supplement Fig. 1**</u> **Effect of FGF-1 on lymphangiogenesis in mouse cornea during HSV-1 infection:** Immunohistochemistry and FACS analysis was carried out to assess if FGF-1 treatment can affect lymphangiogenesis and lymphocyte infiltration in HSV-1 infected mice. B6 mice infected with HSV-1 McKrae ($5X10^5$/eye) were mock treated/ FGF-1 treated (1.6 ng/eye) twice daily from day1 post-infection. (**A**) Panel showing CD31 (endothelial cell adhesion molecule) and (**B**) LYVE-1 (Lymphatic vessel endothelial hyaluronan receptor1) staining in herpes infected mouse cornea mock/treated with FGF-1. Mouse infected with McKrae ($5X10^5$) treated twice a day with FGF-1 (1.6ng/eye) was stained for lymphangiogenesis at day14 PI.

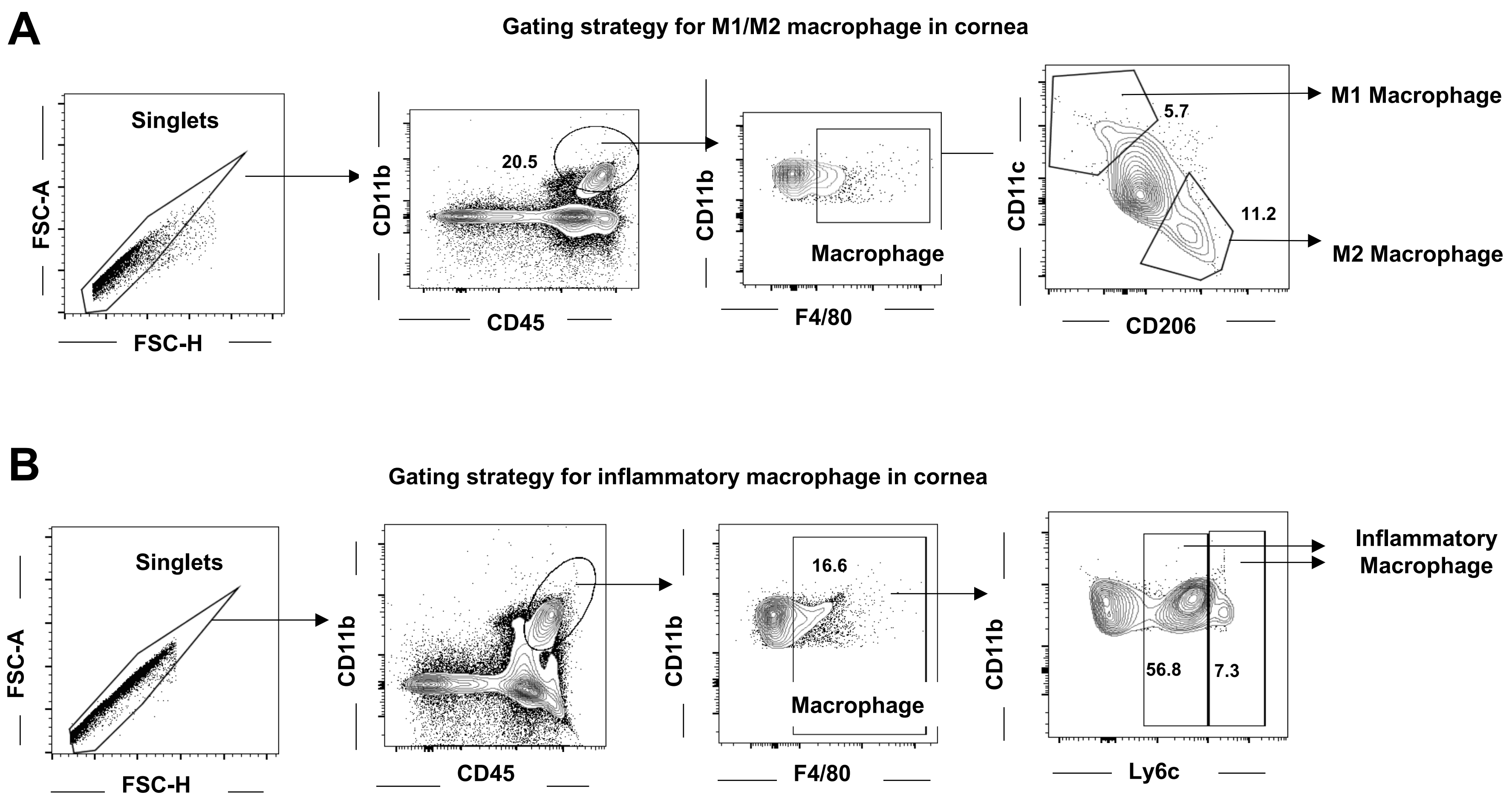

<u>Supplement Fig. 2</u> Panel showing gating strategy for macrophages in mouse corneas: (A) Flow cytometry gating strategy for M1/M2 macrophages in HSV-1 infected cornea. The following gating strategy was used: Lymphocytes were identified by a forward scatter area (FSCA) and side scatter area (SSC-A) gate. Singlets were then selected by plotting forward scatter area (FSC-A) versus forward scatter height (FSC-H). The CD45, CD11b positive cells were then identified, followed by F4/80 positive macrophages. M1 macrophages gated by CD11c expression and M2 macrophages by expression of CD206. **(B)** Flow cytometry gating strategy for inflammatory macrophages in HSV-1 infected cornea. The following gating strategy was used: Lymphocytes were identified by a forward scatter area (FSCA) and side scatter area (SSC-A) gate. Singlets were then selected by plotting forward scatter area (FSC-A) versus forward scatter height (FSC-H). The CD45, CD11b positive cells were then identified, followed by F4/80 positive macrophages. Inflammatory macrophages are then gated by expression levels of Ly6c (medium/intermediate expression).

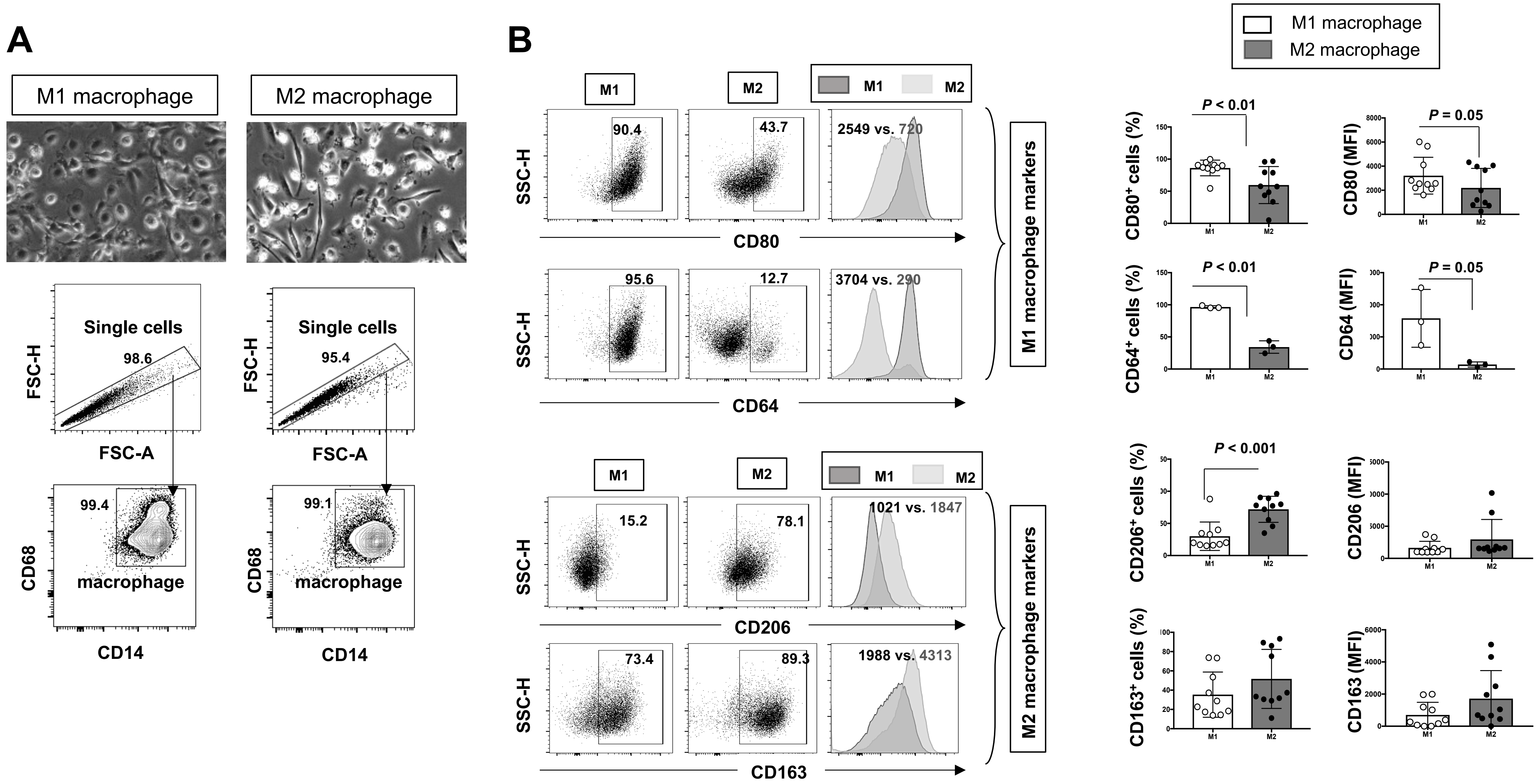

__Supplement Fig. 3__ **Expression of M1 and M2 polarization markers in human monocyte-derived macrophages:** M1- and M2-polarized macrophages were generated from M-CSF-treated primary monocytes stimulated with IFN-g and IL-4, respectively. (**A**) Representative images of M1 and M2 macrophages (top panel). Gating strategy for macrophages as indicated (bottom panel). (**B**) M1 signature markers CD80 & CD64 and M2 signature markers CD163 & CD206 levels were compared by flow cytometry. Dot plots and histograms depict the results obtained in one representative donor; the grey histogram represent the fluorescent profile of M1 macrophages, and the blue histogram represent the fluorescent profile of M1 and M2 macrophages stained with the indicated antibodies. The percentage of positive cells and the MFI (mean fluorescence intensity) is indicated. The graph (right panel) represents mean results from at least three different donors.

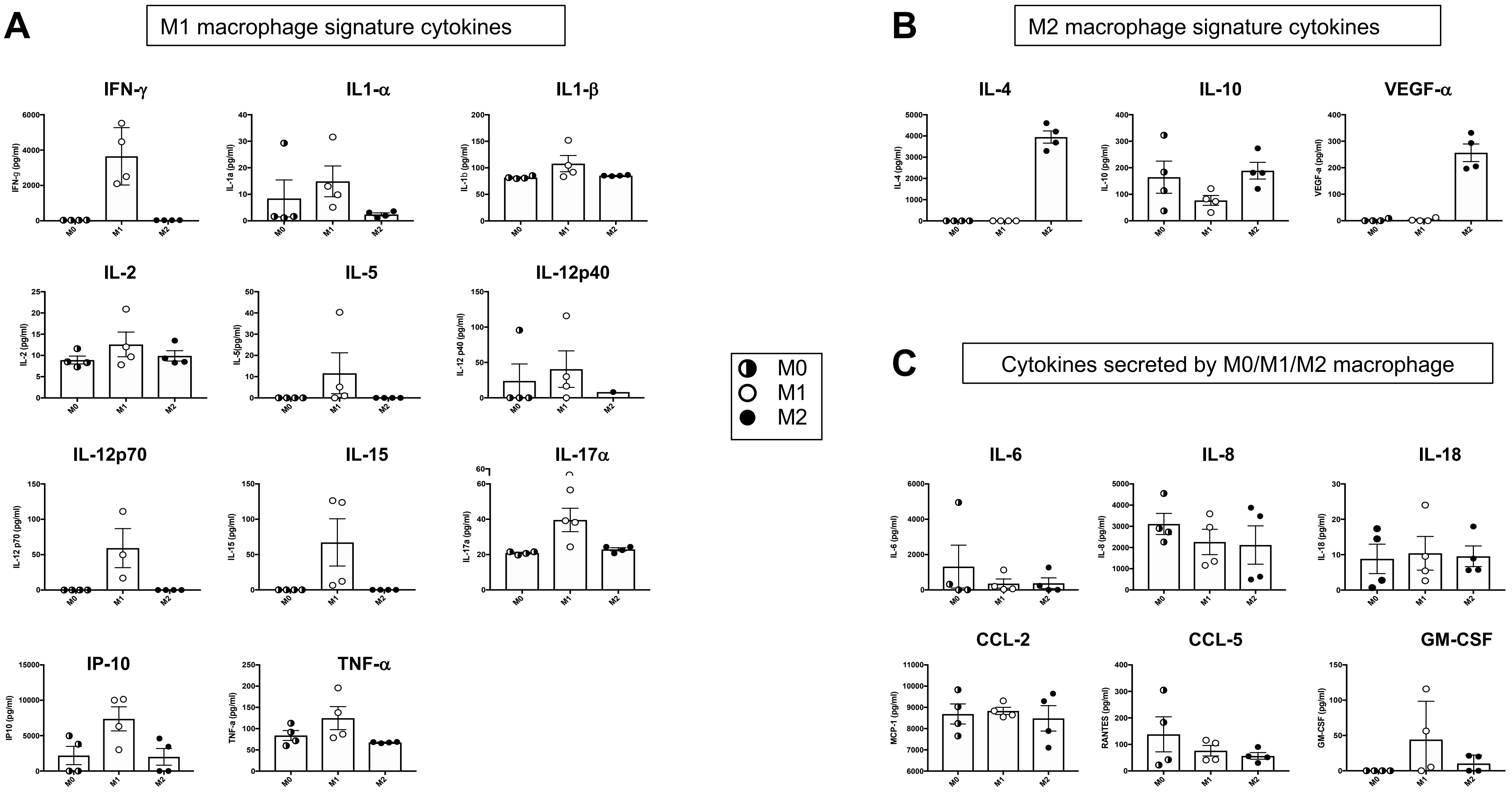

<u>Supplement Fig. 4</u> Secretion of M1 and M2 signature cytokines by M0/M1/M2 human monocyte-derived macrophages: M1- and M2-polarized macrophages were generated from M-CSF-treated primary monocytes stimulated with IFN-γ and IL-4, respectively. (**A**) M1 signature cytokine levels (IFN-γ, IL-1α, IL-1β, IL-2, IL-5, IL-12p40, IL-12 p70, IL-15, I-17, IP-10, TNF-α were estimated by Luminex. (**B**) M2 signature cytokine (IL-4, IL-10, VEGF-α) levels were estimated by Luminex. (**C**) cytokine (IL-6, IL-8, IL-18) and chemokine (CCL2, CCL5, GM-CSF) levels were estimated by Luminex.
